# SSR-stacking: a hybrid GNN–ensemble framework for high-myopia classification under simulated baseline-SE missingness

**DOI:** 10.3389/fpubh.2026.1892477

**Published:** 2026-09-03

**Authors:** Na Zhao, Runze Zheng, Zhaoyu Huang, Cairui Li, Jinhao Lu, Chao Dai, Zhan Tang, Jian Wang

**Affiliations:** 1 School of Software, Yunnan University, Kunming, Yunnan, China; 2 Department of Ophthalmology, People's Hospital of Dali Bai Autonomous Prefecture, Yunnan, China; 3 School of Physics, Zhejiang University, Hangzhou, China; 4 College of Information Engineering and Automation, Kunming University of Science and Technology, Kunming, Yunnan, China

**Keywords:** childhood myopia, explainable machine learning, graph neural networks, high-myopia classification, longitudinal school screening, missing data

## Abstract

The increasing burden of childhood myopia creates a need for screening models that remain informative when routinely collected clinical measurements are incomplete. We propose SSR-Stacking, a hybrid framework integrating Graph Neural Networks (GNN) with Ensemble Learning for classification of final-follow-up high-myopia status under simulated missingness of baseline spherical equivalent (SE). Unlike traditional models, we construct a Social-Environment Graph based on school-class affiliations to represent classroom-level relational structure. Our framework features a Self-Supervised Reconstruction (SSR) mechanism toreconstruct randomly masked baseline SE from neighboring peers and a Meta-Stacking layer to fuse GNN embeddings with diverse ML classifiers (XGBoost, SVM, RF). Evaluated on a longitudinal dataset (*N* = 4, 973) from Binchuan, China, under 50% simulated baseline-SE missingness using classroom-level grouped folds and out-of-fold stacking, SSR-Stacking achieved a ROC-AUC of 0.9184 (95% CI: 0.9062–0.9306), PR-AUC of 0.6120, Recall of 0.8119, Specificity of 0.9851, and F1-score of 0.7506 on unsampled held-out predictions with a natural high-myopia prevalence of 4.06%. These internal results support further evaluation under natural missingness and external validation before clinical deployment.

## 1 Introduction

Large-scale school-based ocular screening is an important component of childhood myopia surveillance (1–4). However, predictive models developed from complete datasets may be difficult to apply when routine measurements are missing. In regional screening programs, biometric and refractive records may be incomplete because of measurement failure, student absence, or heterogeneous administrative systems. Consequently, models that assume a fully observed feature matrix may experience substantial performance degradation when applied under high missingness.

To address missing clinical measurements, conventional analyses commonly use complete-case exclusion or statistical imputation methods such as Multivariate Imputation by Chained Equations (MICE) and K-Nearest Neighbors (KNN) (5, 6). These approaches generally model students independently and do not explicitly represent classroom-level relationships. Epidemiological studies indicate that childhood ocular development is associated with both individual characteristics and contextual exposures, including outdoor time and near-work patterns (7–10). Classroom co-membership may therefore provide additional relational information, although it cannot by itself distinguish shared environment, selection, or other unmeasured factors.

This setting motivates a graph-based computational formulation. Graph neural networks (GNNs), including graph convolutional networks, GraphSAGE, and graph attention networks, learn node representations by propagating information across non-Euclidean neighborhood structures (11–13). Graph-imputation research further suggests that relational dependencies can assist missing-attribute reconstruction when the encoded relationships are informative (14, 15). In school surveillance, classroom affiliation is administratively observable and can be represented as a graph, providing a testable structure for classroom-neighbor message passing.

To address this methodological question, we introduce **SSR-Stacking**, a hybrid graph–ensemble framework for high-myopia classification under simulated baseline-SE sparsity. School surveillance data are represented as a school-class affiliation **Social-Environment Graph**. A **Self-Supervised Reconstruction (SSR)** mechanism uses GNN embeddings to estimate masked baseline SE, and a meta-stacking layer combines graph and tabular classifiers. We internally evaluate the performance of this approach in a longitudinal screening registry of *N* = 4, 973 students from Binchuan, China. By applying random baseline-SE masking from 0% to 80%, with 50% prespecified as the primary benchmarking condition, we assess internal performance under controlled single-variable missingness; broader conclusions require validation under natural multivariable and visit-level missingness.

The primary contributions of this study are as follows:

- **Graph-Based Relational Modeling:** We represent classroom co-membership as a relational association, without assigning causal meaning to the classroom relation, and use it for message passing.
- **Self-Supervised Reconstruction (SSR) Mechanism:** We use a GNN-derived auxiliary task to reconstruct randomly masked baseline SE, with reconstruction error reported for the primary 50% masking condition.
- **Hybrid Meta-Stacking Framework:** We implement a Meta-Stacking layer that combines the structural embeddings of GNNs with the outputs of heterogeneous ML classifiers. Our internal results on the Binchuan dataset (*N* = 4, 973) provide an initial assessment of this integration under simulated baseline-SE missingness.

## 2 Materials and methods

### 2.1 Cohort selection and graph construction

**Figure 1:**
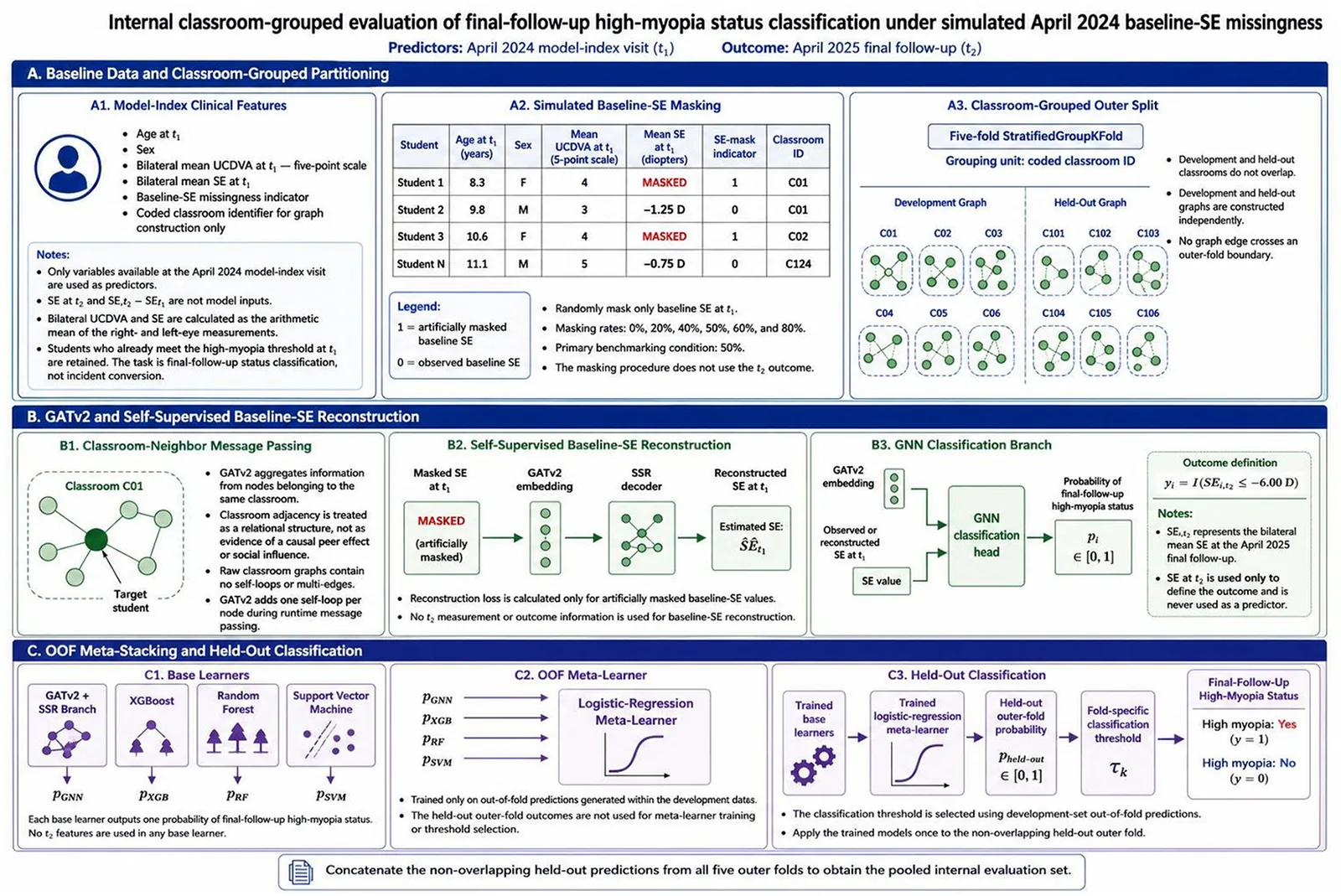
Cohort selection and graph-construction pipeline. The three-step selection process derived the analysis cohort (*N* = 4, 973) from the Binchuan screening registry (*N* = 6, 154). Partition-specific classroom adjacency matrices and April 2024 baseline-only feature matrices were constructed for the prespecified 50% primary baseline-SE masking condition and the 0%–80% masking-gradient analysis.

A prespecified selection pipeline was applied to the Binchuan longitudinal ocular-screening registry to define the analysis cohort and ensure that each included student could be assigned to a classroom graph. As illustrated in Figure 1, the source registry contained 6, 154 student records, which were evaluated using three sequential eligibility and quality-control steps:

- **Tier 1: age eligibility**. Participants were restricted to students aged 6–12 years at the model-index screening in April 2024. This criterion removed 782 records and retained 5,372 candidates. The age range at the 12-month outcome visit consequently extended to 13 years as the cohort aged.
- **Tier 2: pre-index quality control**. To reduce the influence of implausible fluctuations in non-cycloplegic screening measurements, records with a pre-index SE change between April 2023 (*t*_0_) and April 2024 (*t*_1_) exceeding ±4.00 diopters (D) were excluded. An April 2025 (*t*_2_) SE measurement was required to define the outcome, but its value was not used for pre-index quality-control filtering or feature construction. This step removed 285 records.
- **Tier 3: graph eligibility**. A further 114 students were excluded because school, grade, or classroom identifiers were unavailable, preventing assignment to the classroom graph.

These steps yielded a final analysis cohort of *N* = 4, 973 students. The analysis cohort was retained as a balanced complete panel with observed age, sex, bilateral UCDVA, refraction-derived SE, and classroom information at all three visits. Missing records could overlap with the sequential age, quality-control, and graph-eligibility exclusions and were assigned to the first applicable exclusion category; consequently, variable-specific missing counts should not be added to infer additional exclusions. Experimental masks were applied after cohort construction to observed *t*_1_ SE values, enabling reconstruction error to be evaluated against values withheld only during model fitting and inference. Masking rates ranged from 0% to 80%.

For model evaluation, each outer fold generated a baseline-only feature matrix from measurements available at the April 2024 model-index visit and a classroom adjacency matrix constructed independently within the development or held-out partition. No graph edge crossed an outer-fold boundary.

### 2.2 Data collection and clinical measurements

Ocular examinations were performed by trained technicians using a standardized protocol. Uncorrected distance visual acuity (UCDVA) was measured at 5 meters using a logarithmic chart and recorded in the five-point notation used by the screening registry. Non-cycloplegic refraction was measured with an automated infrared refractor (16). Eye-specific spherical equivalent was calculated as *SE* = Sphere+0.5 × Cylinder. For student-level analyses, SE and UCDVA were defined as the arithmetic means of the right- and left-eye values. If either required eye-specific record was unavailable, the corresponding bilateral student-visit field was coded as missing. Throughout the modeling sections, “baseline” refers to the April 2024 model-index visit (*t*_1_); the April 2023 examination (*t*_0_) is termed the pre-index visit. High myopia was defined as student-level mean *SE* ≤ −6.00 D at the April 2025 outcome visit, consistent with the threshold used in international myopia classification guidance (17). Students who already met this threshold at the April 2024 model-index visit were retained. The reported task therefore classifies final-follow-up high-myopia status and includes both students with existing high myopia and students who newly met the threshold; it is not an incident-conversion analysis. Records with a pre-index SE change from April 2023 to April 2024 exceeding ±4.00 D were excluded during pre-index quality control. The April 2025 measurement was used only to define the outcome and was not used for pre-index quality-control filtering, feature construction, reconstruction, or model fitting.

### 2.3 Natural missingness description

Natural missingness was characterized before applying any experimental baseline-SE mask. Item-level missingness was defined as an expected registry field that remained unrecorded among students who attended the corresponding screening wave. Of the 6,154 registered students, 5,972 attended the April 2023 pre-index visit (*t*_0_), 5,509 attended the April 2024 model-index visit (*t*_1_), and 5,170 attended the April 2025 outcome visit (*t*_2_), corresponding to 182, 645, and 984 whole-visit absences, respectively. These wave-specific absences were not mutually exclusive and were not treated as additional exclusion categories. Natural item-level missingness was observed in the registered cohort, whereas the final analysis cohort was a complete panel by design. The simulated experiments therefore evaluated controlled masking of observed *t*_1_ SE within the complete analysis cohort rather than fitting the model directly to naturally incomplete records. Table 1 reports variable-specific natural missingness separately for each screening wave.

**Table 1:** Variable-specific natural missingness across screening waves in the registered and final analysis cohorts.

Screening wave	Cohort group	Attended *N*	Age *n* (%)	Sex *n* (%)	Bilateral UCDVA *n* (%)	Sphere/cylinder *n* (%)	SE *n* (%)	Class information *n* (%)
Pre-index (*t*_0_, 2023)	Registered	5,972	4 (0.07%)	2 (0.03%)	45 (0.75%)	112 (1.88%)	112 (1.88%)	15 (0.25%)
	Final analysis	4,973	0 (0.00%)	0 (0.00%)	0 (0.00%)	0 (0.00%)	0 (0.00%)	0 (0.00%)
Model index (*t*_1_, 2024)	Registered	5,509	3 (0.05%)	2 (0.04%)	52 (0.94%)	108 (1.96%)	108 (1.96%)	18 (0.33%)
	Final analysis	4,973	0 (0.00%)	0 (0.00%)	0 (0.00%)	0 (0.00%)	0 (0.00%)	0 (0.00%)
Outcome (*t*_2_, 2025)	Registered	5,170	5 (0.10%)	4 (0.08%)	48 (0.93%)	100 (1.93%)	100 (1.93%)	12 (0.23%)
	Final analysis	4,973	0 (0.00%)	0 (0.00%)	0 (0.00%)	0 (0.00%)	0 (0.00%)	0 (0.00%)

Denominators for the registered cohort are the students who attended each wave. Sphere and cylinder had identical missing counts because they originated from the same unavailable refraction records; SE was unavailable whenever the required component refraction was unavailable. The final analysis cohort was a balanced complete panel by design, including complete *t*_2_ SE required to define final-follow-up high-myopia status. Natural missingness was observed in the registered cohort, whereas model experiments applied simulated *t*_1_ SE masking only after construction of the complete analysis cohort. Missingness could overlap across variables and waves and should not be summed as mutually exclusive exclusion counts.

### 2.4 Social-environment graph construction

Unlike traditional predictive models that treat students as independent samples, we formulated the prediction task as a node classification problem on a **Social-Environment Graph** G=(V,E).

- **Nodes (**𝒱**):** The revised feature vector was ${\text{x}}_{i}=\left[Age_{t1},Sex,UCDVA_{t1},SE_{t1}^{\text{input}},M_{t1}^{SE}\right]$, where UCDVA and SE are the bilateral student-level means defined above, $SE_{t1}^{\text{input}}$ denotes the observed baseline SE or the masked placeholder supplied to the model, and $M_{t1}^{SE}=1$ indicates an artificially masked baseline-SE value (0 indicates an observed value). All predictive features are restricted to measurements available at the April 2024 index screening (*t*_1_).
- **Edges (**𝓔**):** Connections were established between students belonging to the same school class. This topology encodes classroom co-membership as a relational proxy for potentially shared contextual exposures; it does not by itself identify causal peer or environmental effects.

The final cohort contained 124 classroom clusters with a mean class size of 40.1 students (range, 32–48). Before partitioning, the classroom-clique graph contained 97,304 unique undirected edges, calculated as $E=\overset{124}{\underset{c=1}{\sum }}n_{c}\left(n_{c}-1\right)/2$ from the observed classroom sizes. This corresponded to a mean degree of 39.13, with node degrees ranging from 31 to 47. Raw adjacency matrices contained no self-loops or multiedges. In the PyTorch Geometric representation, each undirected edge was stored in both directions (194,608 directed edge-index entries for the full cohort), and GATv2 added one self-loop per node at runtime. These values describe the full-cohort topology; for every outer fold, separate development and held-out graphs were constructed after classroom-level partitioning, so no message-passing edge crossed a fold boundary.

The temporal prediction boundary was defined with April 2024 as the index time *t*_1_ and April 2025 as the outcome time *t*_2_. The binary target was *y*_*i*_ = *I*(*SE*_*i*,_*t*__2__ ≤ −6.00D). The follow-up progression *SE*_*t*_2__−*SE*_*t*_1__ was excluded from the feature matrix because it is unavailable at *t*_1_ and algebraically contains the outcome measurement.

### 2.5 Spatial autocorrelation analysis

To quantify clustering of SE progression under the classroom adjacency structure, we calculated Global Moran's I. This was a post-follow-up descriptive analysis and was not used to construct predictive features or train the classifier. The weight matrix *W* was defined by *w*_*ij*_ = 1 when students *i* and *j* shared a classroom and *w*_*ij*_ = 0 otherwise. Global Moran's I was computed as:


$$\begin{array}{c} I=\frac{N}{\underset{i}{\sum }\underset{j}{\sum }w_{ij}}\cdot \frac{\underset{i}{\sum }\underset{j}{\sum }w_{ij}\left(y_{i}-ȳ\right)\left(y_{j}-ȳ\right)}{\underset{i}{\sum }{\left(y_{i}-ȳ\right)}^{2}}, \end{array}$$ (1)

where *y*_*i*_ denotes the observed follow-up SE progression of student *i*, ȳ denotes the cohort mean, and *N* is the number of students. This analysis tests whether progression values are clustered under classroom-based adjacency; it does not identify a causal classroom effect. Neither *y*_*i*_ nor any quantity derived from *SE*_*t*_2__ was provided to the prediction models.

### 2.6 Proposed SSR-stacking framework

**Figure 2:**
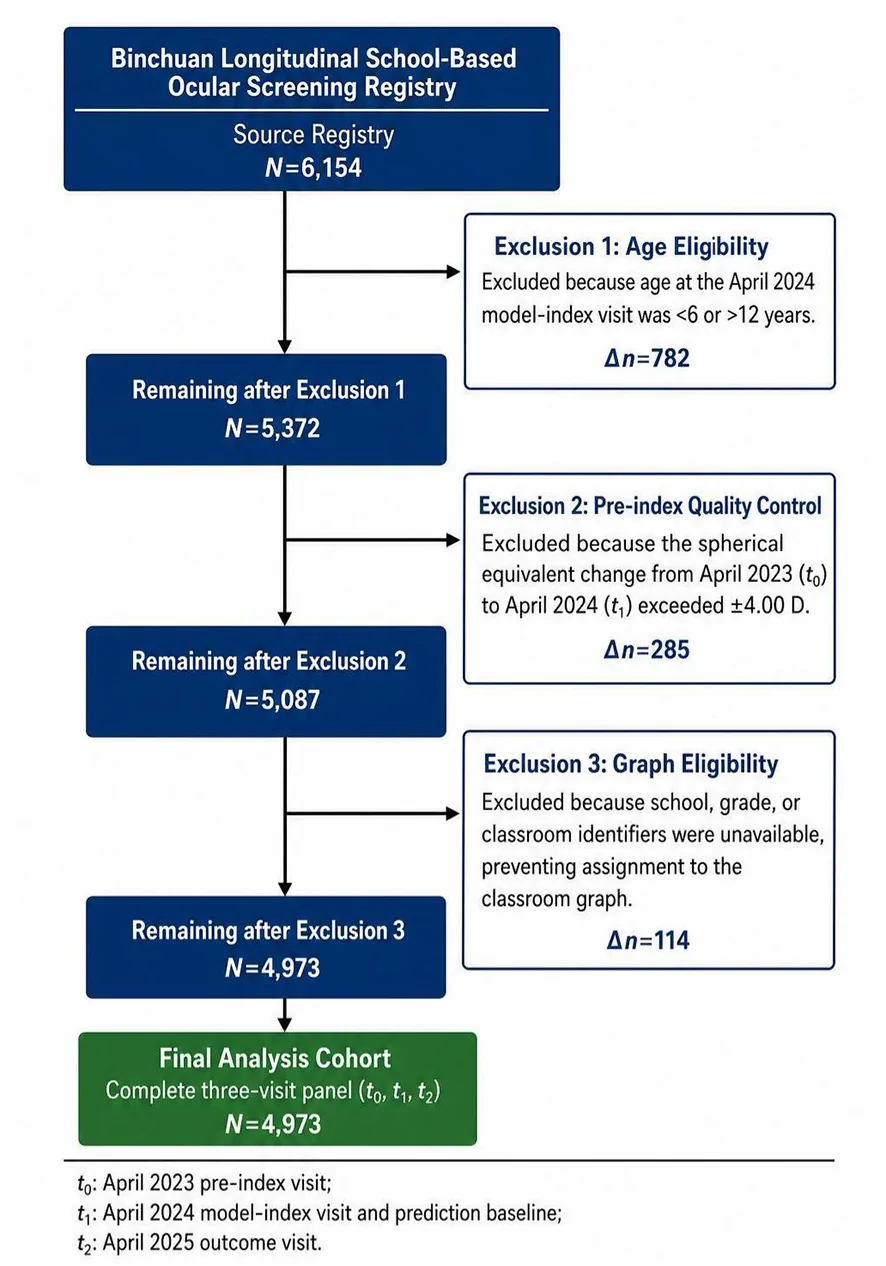
Overall architecture of the proposed SSR-Stacking framework. The workflow integrates social-environment graph construction, GNN-based self-supervised reconstruction, heterogeneous machine learning classifiers, and a meta-stacking layer for high-myopia classification under simulated baseline-SE missingness. The SSR module uses attention-weighted classroom-neighbor embeddings to reconstruct masked baseline SE; under the prespecified 50% masking condition, its held-out reconstruction MAE was 0.32 D.

The **SSR-Stacking** framework combines a relational GNN branch, baseline-SE reconstruction, tabular classifiers, and an OOF stacking layer. The overall architecture is illustrated in Figure 2.

#### 2.6.1 Hybrid-GNN branch with SSR mechanism

We implemented a Graph Attention Network (GATv2) branch to perform message passing and learn relative weights across classroom neighbors. In the social-environment graph G, the contribution of neighbor $j\in N_{i}$ to target student *i* is represented by an attention score:


$$\begin{array}{c} e_{ij}={\vec{a}}^{T}\mathrm{LeakyReLU}\left(\text{W}\cdot \left[{\text{h}}_{i}∥{\text{h}}_{j}\right]\right), \end{array}$$ (2)

where [**h**_*i*_∥**h**_*j*_] denotes concatenated target- and neighbor-node representations, **W**∈ℝ^*d*′ × *d*^ is a learnable transformation, and $\vec{a}\in {\mathbb{R}}^{2d^{\prime }}$ is the attention vector. LeakyReLU used a negative slope of 0.2. The normalized attention weight is computed as:


$$\begin{array}{c} \alpha_{ij}={\mathrm{softmax}}_{j}\left(e_{ij}\right)=\frac{\exp\left(e_{ij}\right)}{\underset{k\in N_{i}}{\sum }\exp\left(e_{ik}\right)}. \end{array}$$ (3)

The enhanced topological representation of node *i* is then aggregated as:


$$\begin{array}{c} {\text{h}}_{i}^{\prime }=\sigma \left(\underset{j\in N_{i}}{\sum }\alpha_{ij}\text{W}{\text{h}}_{j}\right), \end{array}$$ (4)

where σ(·) denotes the nonlinear activation function. To address missing baseline SE, the **Self-Supervised Reconstruction (SSR)** module maps the GNN-derived latent representation to a scalar SE estimate through a two-layer MLP decoder:


$$\begin{array}{c} {\hat{SE}}_{i,t_{1}}={\mathrm{MLP}}_{decoder}\left({\text{h}}_{i}^{\prime }\right)={\text{w}}_{2}^{⊤}\phi \left({\text{W}}_{1}{\text{h}}_{i}^{\prime }+{\text{b}}_{1}\right)+b_{2}. \end{array}$$ (5)

The decoder uses a hidden dimension of *d*_*hidden*_ = 128 with ReLU activation ϕ(·) in the hidden layer. The scalar output uses a linear activation so that baseline SE is reconstructed in diopters. The GNN-branch training objective combines its supervised classification loss with self-supervised baseline-SE recovery:


$$\begin{array}{c} L_{total}=L_{CE}\left(y,{ŷ}_{\text{GNN}}\right)+\lambda \cdot L_{SSR}\left(SE_{mask},\hat{SE}\right), \end{array}$$ (6)

where $L_{CE}$ is the cross-entropy loss for GNN-branch classification and λ = 0.5 balances classification and reconstruction objectives. The logistic-regression meta-learner is not optimized by this loss and is fitted subsequently using only OOF base-learner predictions. The SSR loss is defined only over masked nodes:


$$\begin{array}{c} L_{SSR}=\frac{1}{\left|V_{mask}\right|}\underset{i\in V_{mask}}{\sum }{\left(SE_{i,t_{1}}-{\hat{SE}}_{i,t_{1}}\right)}^{2}. \end{array}$$ (7)

This design encourages the network to learn attention weights α_*ij*_ that identify neighboring information useful for reconstructing masked baseline SE. The detailed procedure is described in Algorithm 1.

Algorithm 1 GNN-Branch Self-Supervised Reconstruction and OOF Meta-Stacking 
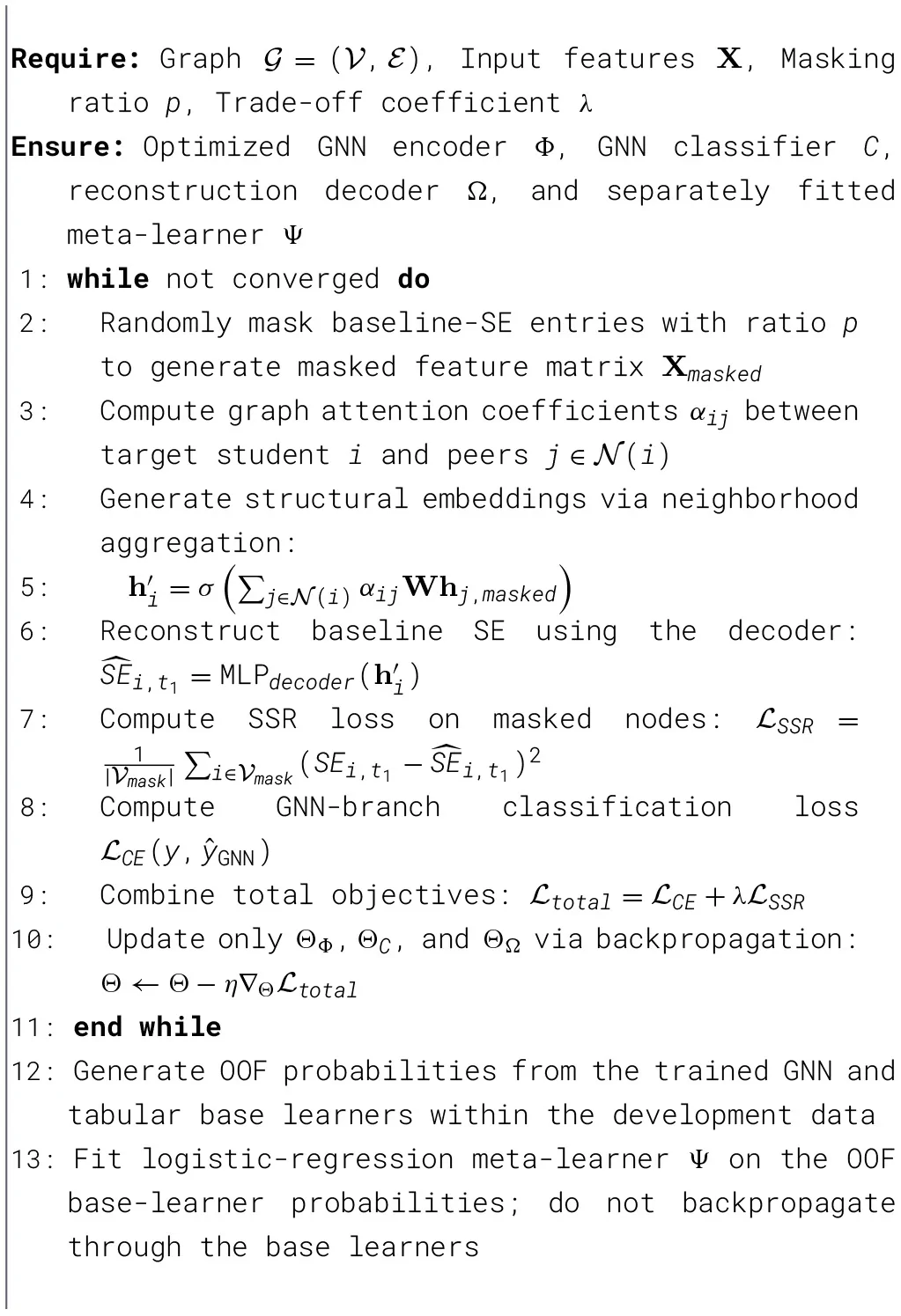

#### 2.6.2 Meta-stacking ensemble layer

Predicted probabilities from the GNN branch, XGBoost, Random Forest, and SVM were combined by a logistic-regression meta-learner (18–21). For each outer fold, the base learners generated OOF predictions within the development data. The meta-learner was fitted only on these OOF probabilities and was then applied to base-learner predictions from the held-out outer fold; it was not jointly optimized with the GNN by backpropagation. The classification inputs were restricted to demographic and ocular measurements available at the April 2024 index screening (*t*_1_). The model estimates final-follow-up high-myopia status at April 2025 (*t*_2_). Continuous *SE*_*t*_2__, *SE*_*t*_2__−*SE*_*t*_1__, and all other post-index measurements were excluded from predictor construction, reconstruction inputs, stacking inputs, and candidate model inputs during hyperparameter selection. Within each outer fold, the binary *t*_2_ outcome was used as a supervised label only in the development partition; held-out outcomes remained unavailable until final evaluation.

### 2.7 Threshold selection and calibration assessment

Classification thresholds were selected separately within each outer fold using only the corresponding training-fold OOF predictions. The primary rule selected the threshold that maximized specificity while maintaining sensitivity at or above 0.80; the resulting threshold was then applied without modification to the held-out fold. The median threshold across the five outer folds was 0.082. Probability calibration was summarized from the concatenated classroom-grouped held-out predictions using the Brier score, calibration intercept, calibration slope, and observed-to-expected ratio. The intercept and slope were defined through logistic recalibration, $\mathrm{logit}\left(Y\right)=a+b\mathrm{logit}\left(\hat{p}\right)$, with ideal values of 0 and 1, respectively. The observed-to-expected ratio was defined as the observed prevalence divided by the mean predicted probability. Net benefit at each reported operating threshold was calculated as [*TP*/*N*−(*FP*/*N*){*p*_*t*_/(1−*p*_*t*_)}] × 1, 000.

### 2.8 Paired classroom-cluster uncertainty analysis

Model-specific ROC-AUC confidence intervals were estimated using 1,000 classroom-level cluster-bootstrap resamples, with all students from a sampled classroom retained together. Prediction-level paired bootstrap intervals were not available for every between-model metric or masking level. Accordingly, between-model differences are reported as descriptive effect estimates rather than formal significance tests.

### 2.9 Baseline-SE missingness gradient analysis

Baseline SE was masked at rates of 0%, 20%, 40%, 50%, 60%, and 80%. The classroom-grouped outer folds were held fixed across masking conditions, and the same within-fold masks were used for competing models at each missingness level. Metrics were calculated from concatenated held-out predictions without test-set resampling, preserving the natural high-myopia prevalence. Gradient comparisons are reported as point estimates unless a prediction-level classroom-cluster bootstrap interval is explicitly provided.

### 2.10 Ablation analysis setup

Five configurations were compared under 50% baseline-SE missingness: (1) ML stacking without graph inputs; (2) GATv2 without SSR or stacking; (3) GATv2 plus OOF stacking without SSR; (4) GATv2 plus SSR without stacking; and (5) the full SSR-Stacking framework. All configurations used the same baseline-only predictors, classroom-grouped folds, held-out evaluation protocol, and threshold-selection procedure. Because repeated-seed and paired interaction estimates were unavailable, ablation results are reported as point estimates and are not described as statistical synergy.

### 2.11 Reconstruction accuracy assessment

Reconstruction accuracy was evaluated exclusively on artificially masked held-out samples under the prespecified 50% baseline-SE masking condition. The true baseline SE was unavailable during model fitting, hyperparameter selection, and inference and was accessed only after prediction for error calculation. Mean, MICE, MissForest, GAIN, and GRAPE comparators were fitted using the corresponding training folds only. Reconstruction performance was summarized using MAE, RMSE, signed bias defined as reconstructed minus true SE, and the proportion of absolute errors not exceeding 0.50 D. Paired student-level values required for a Bland–Altman plot and limits of agreement were not available in the aggregate reporting export; therefore, no Bland–Altman result is reported.

### 2.12 Secondary robustness, interpretability, and school-level evaluation

Feature attribution was restricted to the XGBoost tabular branch and was not interpreted as a global explanation of the complete SSR-Stacking framework. TreeSHAP values were calculated separately for each outer-fold model on its corresponding held-out samples and were then concatenated. The explainer received exactly the inference-time features: age, sex, UCDVA, reconstructed or available baseline SE, and the baseline-SE missing indicator. SHAP values were expressed on the log-odds scale and were interpreted as predictive attribution rather than causal effect. Age-stratified evaluation used three prespecified brackets (6–8, 9–10, and 11–12 years) selected to retain sufficient positive-event counts. Within-region school-level generalizability was assessed through leave-one-school-out internal–external validation. For each iteration, standardization, graph construction, reconstruction, hyperparameter optimization, stacking, and threshold selection used development schools only, and the held-out school graph was constructed independently. School A–D are anonymized identifiers for the four participating schools.

### 2.13 Implementation details and hyperparameters

The framework was implemented in PyTorch 2.1 with PyTorch Geometric. The GNN branch used two GATv2 layers, eight attention heads, and a latent dimension of 128. The reconstruction-loss weight was λ = 0.5. XGBoost used 100 estimators with maximum depth 6, and Random Forest used 500 trees. The GNN was trained for 200 epochs with Adam (*LR* = 10^−3^) on an NVIDIA RTX 4090. MICE, MissForest, GAIN, GRAPE, standalone GCN, and standalone GATv2 were adapted to the same input format. Every classifier received the same five-variable baseline-only feature set $\left[Age_{t_{1}},Sex,UCDVA_{t_{1}},{\tilde{SE}}_{t_{1}},M_{t_{1}}^{SE}\right]$, where ${\tilde{SE}}_{t_{1}}$ denotes the observed SE when available or the fold-specific reconstructed SE when artificially masked, and $M_{t_{1}}^{SE}$ is the corresponding masking indicator; no model accessed follow-up SE or derived progression variables as predictors. Outer partitions were generated from classroom identifiers using StratifiedGroupKFold(n_splits=5, shuffle=True, random_state=42), so that all students from one classroom remained in the same fold while the positive proportion was approximately balanced. Random seed 42 was also used for simulated masking and model-weight initialization; graph topology itself was deterministic from classroom identifiers. Graphs and message-passing edges were constructed separately within development and held-out partitions. The stacking meta-learner was trained exclusively on development-data OOF predictions. For each outer fold, the grouped split was defined before simulated masking, and masks were generated independently within the development and held-out partitions. Reconstruction losses did not access held-out labels or the unmasked held-out SE values used later for error calculation. Final point estimates were calculated from concatenated, non-overlapping held-out predictions at the natural outcome prevalence. Classroom-level cluster bootstrap resampling was used only to estimate confidence intervals.

## 3 Results

### 3.1 Cohort characteristics and graph motivation

Before evaluating predictive performance, we characterized the longitudinal cohort (*N* = 4, 973) and assessed classroom-level clustering as motivation for the graph representation.

#### 3.1.1 Population-level refractive trajectory

As summarized in Table 2, bilateral mean SE shifted from −0.77 ± 1.47 D at the April 2023 pre-index visit to −1.37 ± 2.10 D at the April 2025 outcome visit (*P* < 0.001). Mean UCDVA scores also declined over the same period.

**Table 2:** Longitudinal ocular characteristics at the pre-index, model-index, and outcome visits (*N* = 4, 973).

Clinical metrics	Pre-index (2023, *t*_0_)	Model index (2024, *t*_1_)	Outcome (2025, *t*_2_)	Net change	Annualized aggregate change	*P*-value^*^
1. Refractive components (D)						
Bilateral Mean Spherical Equivalent (SE)	−0.77 ± 1.47	−1.30 ± 1.93	−1.37 ± 2.10	−0.60	−0.30	< 0.001
Right Eye Sphere (S)	−0.43 ± 1.49	−1.08 ± 1.90	−1.19 ± 2.04	−0.76	–	< 0.001
Left Eye Sphere (S)	−0.33 ± 1.45	−0.90 ± 1.85	−0.98 ± 1.95	−0.65	–	< 0.001
Bilateral Mean Cylinder (Astigmatism)	−0.78 ± 0.71	−0.62 ± 0.69	−0.57 ± 0.75	+0.21	–	< 0.05
2. Visual function (UCDVA)						
Right Eye (5-point chart score)	4.81 ± 0.32	4.70 ± 0.39	4.67 ± 0.41	−0.14	–	< 0.001
Left Eye (5-point chart score)	4.81 ± 0.32	4.72 ± 0.38	4.69 ± 0.40	−0.12	–	< 0.001
Visual Impairment Rate (< 5.0)	42.3%	57.1%	59.2%	+16.9%	–	< 0.001
3. Myopia progression (%)						
Overall Prevalence	42.28%	59.80%	52.32%^†^	+10.04%	–	< 0.001
Mild Myopia Prevalence	34.02%	43.60%	32.99%	–	–	–
Moderate Myopia Prevalence	7.15%	13.44%	15.26%	+8.11%	–	–
High Myopia Prevalence	1.11%	2.76%	4.06%	+2.95%	–	–
4. Demographic slice						
Age Range by Study Design (Years)	5–11	6–12	7–13	+2.00 y	+1.00 y	–
Gender (Male Ratio)	48.4%	48.4%	48.4%	–	–	–

^*^ Repeated-measures ANOVA for continuous variables; Cochran's Q test for repeated binary variables. ^†^ Visit-specific prevalence reflects non-cycloplegic threshold classification within the same analytic cohort; individual classifications may cross category thresholds between annual visits. Student-level SE was the arithmetic mean of right- and left-eye SE. The final analysis cohort had complete core variables at all three visits by design (Table 1). Prevalence changes are percentage-point differences.

The aggregate 24-month mean SE change was −0.60 D, corresponding to approximately −0.30 D/year when annualized from the displayed visit means. This descriptive trajectory was not used as a predictor in the April 2024 index-based classification model.

#### 3.1.2 Classroom-level clustering

We calculated the intraclass correlation coefficient (ICC) and Global Moran's I for post-follow-up SE progression under classroom grouping.

The ICC was 0.24 (*P* < 0.01), and Global Moran's I was 0.21, indicating classroom-level statistical clustering. These statistics do not establish that classroom exposure caused 24% of progression and cannot distinguish shared environment from selection, demographic composition, or unmeasured factors. The observed clustering provides empirical motivation for evaluating a classroom graph, which is treated as a relational structure rather than a causal exposure measure.

### 3.2 Predictive performance under simulated missingness

We benchmarked **SSR-Stacking** under a simulated **50% baseline-SE missing rate**, defined as the primary experimental stress-test condition. Comparators included traditional ML, imputation-plus-ML pipelines, standalone graph models, and graph-imputation methods.

As shown in Table 3, traditional ML models had lower sensitivity under simulated baseline-SE missingness. SVM, RF, and XGBoost achieved Recall values of 0.4208, 0.5099, and 0.5644, respectively. MissForest + XGBoost increased Recall to 0.6485, while standalone GATv2 reached 0.6832. GAIN and GRAPE reached Recall values of 0.7129 and 0.7277, respectively. At the fold-specific thresholds, SSR-Stacking identified 164 of 202 positive cases and produced 71 false-positive classifications, corresponding to Recall 0.8119, Specificity 0.9851, Precision 0.6979, balanced accuracy 0.8985, and F1-score 0.7506. Its ROC-AUC was 0.9184 (95% CI: 0.9062–0.9306) and its PR-AUC was 0.6120. Formal paired model-comparison intervals are not reported; therefore, comparisons are described using effect estimates rather than unverified significance claims.

**Table 3:** Comprehensive performance benchmarking under 50% simulated baseline-SE missingness using classroom-grouped held-out predictions.

Category	Algorithm/framework	ROC-AUC (95% CI)	PR-AUC	Accuracy	BACC^*a*^	Precision	Recall (Sens.)	Specificity	F1-Score
Traditional ML	Support Vector Machine (SVM)	0.7425 (0.7214–0.7636)	0.1845	0.9210	0.6815	0.2355	0.4208	0.9422	0.3020
	Random Forest (RF)	0.7812 (0.7610–0.8014)	0.2450	0.9385	0.7333	0.3323	0.5099	0.9566	0.4023
	XGBoost	0.8045 (0.7852–0.8238)	0.2912	0.9461	0.7633	0.3878	0.5644	0.9623	0.4597
Imputation + ML	MICE + XGBoost	0.8265 (0.8080–0.8450)	0.3415	0.9540	0.7911	0.4509	0.6139	0.9684	0.5199
	MissForest + XGBoost	0.8420 (0.8241–0.8599)	0.3890	0.9610	0.8114	0.5157	0.6485	0.9742	0.5746
Graph baselines	Standalone GCN (Mean Imp.)	0.8390 (0.8212–0.8568)	0.3750	0.9590	0.8032	0.4961	0.6337	0.9728	0.5565
	Standalone GATv2 (Zero Imp.)	0.8588 (0.8415–0.8761)	0.4285	0.9654	0.8303	0.5610	0.6832	0.9774	0.6161
Graph imputation	GAIN	0.8745 (0.8580–0.8910)	0.4680	0.9694	0.8466	0.6050	0.7129	0.9803	0.6545
	GRAPE	0.8812 (0.8650–0.8974)	0.4915	0.9714	0.8547	0.6282	0.7277	0.9818	0.6743
**Ours**	**SSR-stacking (full framework)**	**0.9184 (0.9062–0.9306)**	**0.6120**	**0.9781**	**0.8985**	**0.6979**	**0.8119**	**0.9851**	**0.7506**

Performance was evaluated using concatenated held-out predictions from classroom-level grouped folds without test-set resampling (*N*_pos_ = 202, *N*_neg_ = 4, 771; prevalence = 4.06%). Fold-specific thresholds were selected from the corresponding training-fold OOF predictions by maximizing specificity subject to sensitivity ≥0.80; the median threshold was 0.082. ROC-AUC confidence intervals were estimated using 1,000 classroom-level cluster-bootstrap resamples. ^*a*^BACC denotes balanced accuracy: (Sensitivity+Specificity)/2. Bold values indicate the best performance among evaluated models/proposed framework.

**Benchmarking against comparison methods**. We compared **SSR-Stacking** with traditional ML classifiers, imputation-enhanced ML pipelines, standalone GNN architectures, and graph-imputation methods. All models were evaluated under 50% simulated baseline-SE missingness using the same classroom-grouped folds and held-out evaluation protocol.

As shown in Table 3, the performance estimates followed a methodological hierarchy. Traditional ML was most affected by missing baseline SE, imputation-enhanced ML improved performance, and graph-based methods produced higher Recall and PR-AUC values. GRAPE was the strongest non-proposed comparator, with a ROC-AUC of 0.8812 (95% CI: 0.8650–0.8974), PR-AUC of 0.4915, Recall of 0.7277, and F1-score of 0.6743. Relative to GRAPE, SSR-Stacking showed absolute increases of 0.0372 in ROC-AUC, 0.1205 in PR-AUC, 0.0842 in Recall, and 0.0763 in F1-score. These point-estimate differences are compatible with complementary contributions from reconstruction and heterogeneous classifier integration, but they do not establish clinical utility, statistical superiority, or transportability.

**Figure 3:**
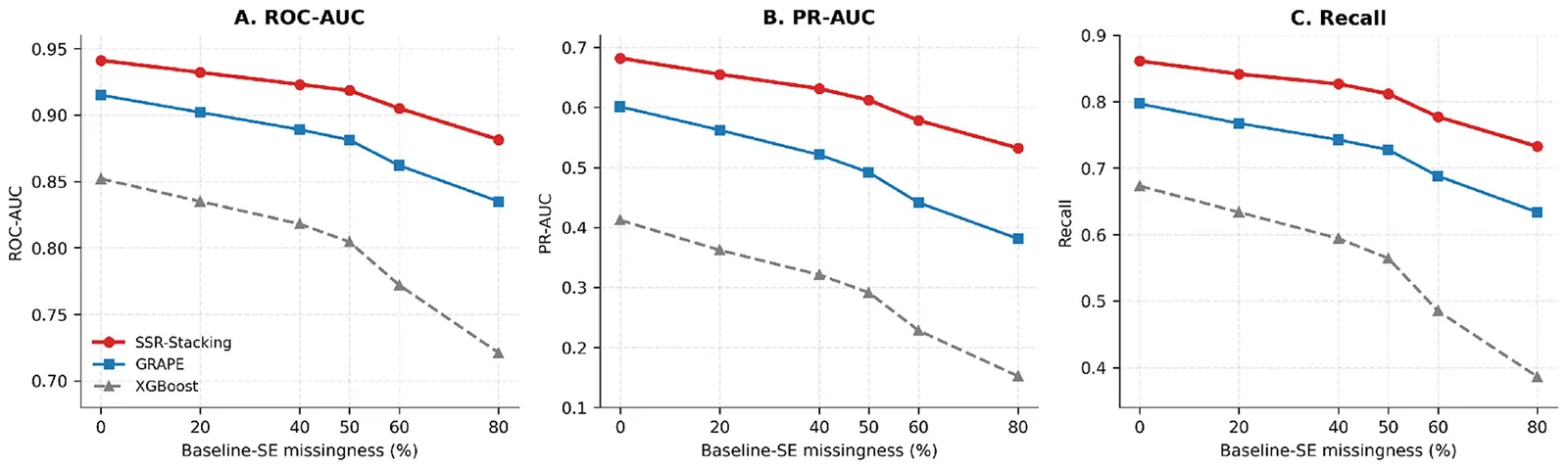
Performance across simulated baseline-SE missingness levels. ROC-AUC, PR-AUC, and Recall are shown for SSR-Stacking, GRAPE, and XGBoost using fixed classroom-grouped folds. Lines connect point estimates and should not be interpreted as fitted degradation functions. Confidence bands are omitted because prediction-level paired classroom-cluster bootstrap intervals were not available for every masking condition. **(A)** ROC-AUC, **(B)** PR-AUC, and **(C)** Recall.

Performance across increasing masking levels. Across baseline-SE masking rates from 0% to 80%, performance decreased for all three representative frameworks (Figure 3, Table 4). SSR-Stacking ROC-AUC decreased from 0.9412 to 0.8815, PR-AUC from 0.6820 to 0.5322, and Recall from 0.8614 to 0.7327. The PR-AUC difference between SSR-Stacking and GRAPE increased from 0.0810 at 0% masking to 0.1512 at 80% masking. These are descriptive point-estimate comparisons; paired classroom-cluster confidence intervals for the between-model differences were not available.

**Table 4:** Performance benchmark across the complete baseline-SE missingness gradient (0% to 80%).

Missingness	Category	Algorithm	ROC-AUC (95% CI)	PR-AUC	Recall	Specificity	Precision	F1-Score
0%	ML	XGBoost	0.8520 (0.8350–0.8690)	0.4120	0.6733	0.9740	0.5231	0.5887
	Graph	GRAPE	0.9150 (0.9010–0.9290)	0.6010	0.7970	0.9870	0.7220	0.7576
	**Ours**	**SSR-Stacking**	**0.9412 (0.9300–0.9524)**	**0.6820**	**0.8614**	**0.9891**	**0.7699**	**0.8131**
20%	ML	XGBoost	0.8350 (0.8170–0.8530)	0.3620	0.6337	0.9690	0.4638	0.5356
	Graph	GRAPE	0.9020 (0.8870–0.9170)	0.5620	0.7673	0.9849	0.6828	0.7226
	**Ours**	**SSR-Stacking**	**0.9320 (0.9200–0.9440)**	**0.6550**	**0.8416**	**0.9881**	**0.7489**	**0.7925**
40%	ML	XGBoost	0.8180 (0.8000–0.8360)	0.3210	0.5941	0.9650	0.4181	0.4908
	Graph	GRAPE	0.8890 (0.8730–0.9050)	0.5210	0.7426	0.9830	0.6494	0.6928
	**Ours**	**SSR-Stacking**	**0.9230 (0.9110–0.9350)**	**0.6310**	**0.8267**	**0.9862**	**0.7167**	**0.7678**
50%	ML	XGBoost	0.8045 (0.7852–0.8238)	0.2912	0.5644	0.9623	0.3878	0.4597
	Graph	GRAPE	0.8812 (0.8650–0.8974)	0.4915	0.7277	0.9818	0.6282	0.6743
	**Ours**	**SSR-Stacking**	**0.9184 (0.9062–0.9306)**	**0.6120**	**0.8119**	**0.9851**	**0.6979**	**0.7506**
60%	ML	XGBoost	0.7720 (0.7510–0.7930)	0.2280	0.4851	0.9510	0.2952	0.3670
	Graph	GRAPE	0.8620 (0.8440–0.8800)	0.4410	0.6881	0.9780	0.5697	0.6233
	**Ours**	**SSR-Stacking**	**0.9050 (0.8920–0.9180)**	**0.5780**	**0.7772**	**0.9830**	**0.6597**	**0.7136**
80%	ML	XGBoost	0.7210 (0.6980–0.7440)	0.1520	0.3861	0.9279	0.1848	0.2500
	Graph	GRAPE	0.8350 (0.8160–0.8540)	0.3810	0.6337	0.9711	0.4812	0.5470
	**Ours**	**SSR-Stacking**	**0.8815 (0.8660–0.8970)**	**0.5322**	**0.7327**	**0.9790**	**0.5968**	**0.6578**

Metrics were calculated at the natural outcome prevalence (*N*_pos_ = 202, *N*_neg_ = 4, 771). Precision and F1-score were checked against the integer confusion matrices implied by Recall and Specificity. ROC-AUC confidence intervals summarize individual models. Bold values indicate the best performance among evaluated models/proposed framework.

### 3.3 Ablation and interpretability analysis

Under the revised 50% masking protocol, the GATv2-only configuration achieved PR-AUC 0.4285 and Recall 0.6832 (Table 5). Adding OOF stacking without SSR increased PR-AUC by 0.0610 and Recall by 0.0396. Adding SSR without stacking increased PR-AUC by 0.1085 and Recall by 0.0792 (7.92 percentage points). The full framework achieved PR-AUC 0.6120 and Recall 0.8119, absolute increases of 0.1835 and 0.1287 (12.87 percentage points), respectively, over GATv2 only. Because repeated-run uncertainty and a formal interaction estimate were unavailable, these results are interpreted as descriptive component contributions rather than statistical synergy.

**Table 5:** Ablation analysis under 50% simulated baseline-SE missingness.

Configuration	Graph	SSR loss	Stacking	ROC-AUC	PR-AUC	Recall	F1-Score	ΔPR-AUC^*a*^
ML Stacking	No	No	Yes	0.8152	0.3120	0.5842	0.4816	−0.1165
GATv2 only	Yes	No	No	0.8588	0.4285	0.6832	0.6161	Reference
GATv2 + Stacking	Yes	No	Yes	0.8780	0.4895	0.7228	0.6697	+0.0610
GATv2 + SSR	Yes	Yes	No	0.8954	0.5370	0.7624	0.7032	+0.1085
Full SSR-stacking	Yes	Yes	Yes	0.9184	0.6120	0.8119	0.7506	+0.1835

^*a*^Absolute PR-AUC difference relative to GATv2 only. Values are point estimates from the common classroom-grouped evaluation protocol. Bold values indicate the best performance among evaluated models/proposed framework.

#### 3.3.1 Reconstruction fidelity

**Figure 4:**
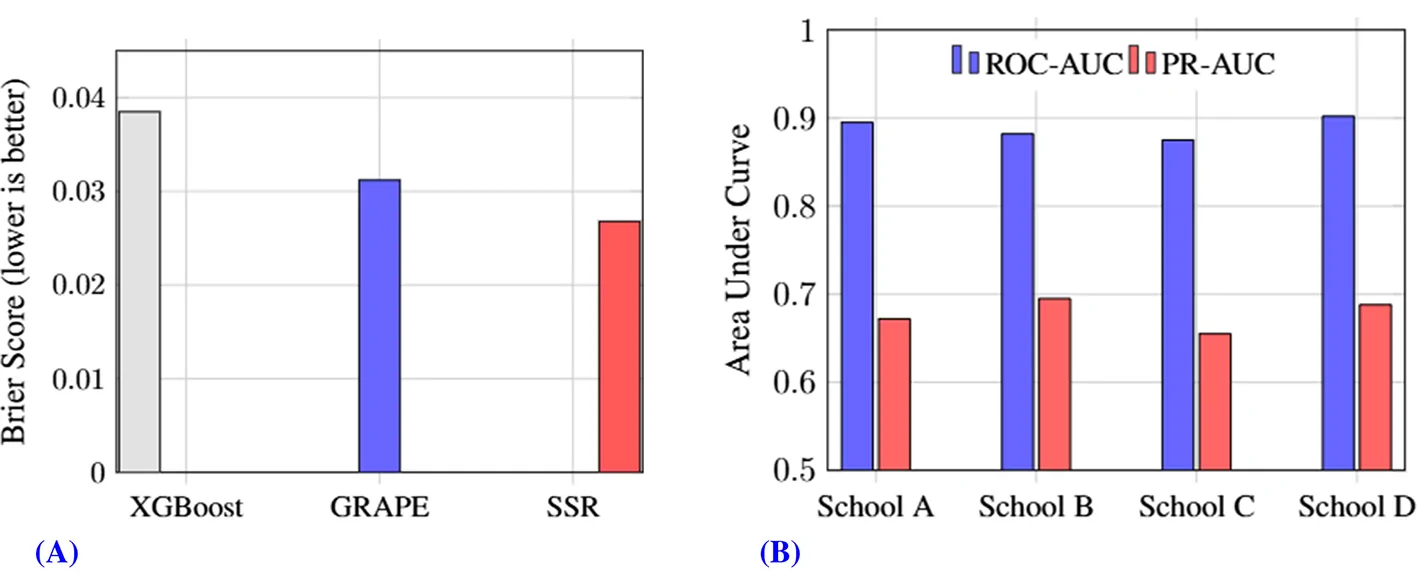
Aggregate validation summaries at 50% simulated baseline-SE missingness. **(A)** compares reported reconstruction MAE and RMSE. **(B)** displays reported mean absolute SHAP magnitudes for the held-out XGBoost branch and does not represent attribution direction or distribution. **(C)** presents age-subgroup PR-AUC point estimates without confidence intervals; the dashed line marks the overall-cohort estimate.

The reconstruction evaluation used artificially masked held-out samples under the prespecified 50% baseline-SE masking condition. All comparator imputers were fitted using development-fold data only, and bias was defined as reconstructed minus true SE. SSR achieved the lowest reported MAE (0.32 D) and RMSE (0.45 D), with a bias of 0.01 D and 89.3% of absolute errors within 0.50 D (Table 6, Panel A; Figure 4, Panel A). A Bland–Altman plot is not presented because the aggregate reporting export did not retain the paired student-level values required to display the point distribution and calculate limits of agreement.

**Table 6:** Held-out reconstruction error and leakage-controlled XGBoost SHAP results.

Panel A. Reconstruction error metrics under 50% baseline-SE masking				
Method	MAE (D)	RMSE (D)	Bias (D)^*a*^	|Error| ≤ 0.50 D
Mean	0.92	1.15	0.12	34.5%
MICE	0.78	0.95	0.08	48.2%
MissForest	0.71	0.88	0.06	55.4%
GAIN	0.65	0.81	0.05	62.1%
GRAPE	0.58	0.74	0.03	68.7%
SSR	0.32	0.45	0.01	89.3%
**Panel B. SHAP feature-importance ranking for the held-out XGBoost branch**				
**Feature**			**Mean absolute SHAP**	**Rank**
Reconstructed/available *SE*_*t*1_			1.45	1
*UCDVA*_*t*1_			0.82	2
*Age*_*t*1_			0.35	3
Sex			0.12	4
Baseline-SE missing indicator			0.08	5

^*a*^Bias is reconstructed minus true baseline SE. Reconstruction metrics use only artificially masked held-out samples. SHAP values were calculated fold-wise on held-out samples and are reported on the log-odds scale. Bold values indicate the best performance among evaluated models/proposed framework.

#### 3.3.2 Component-level interpretability

**Figure 5:**
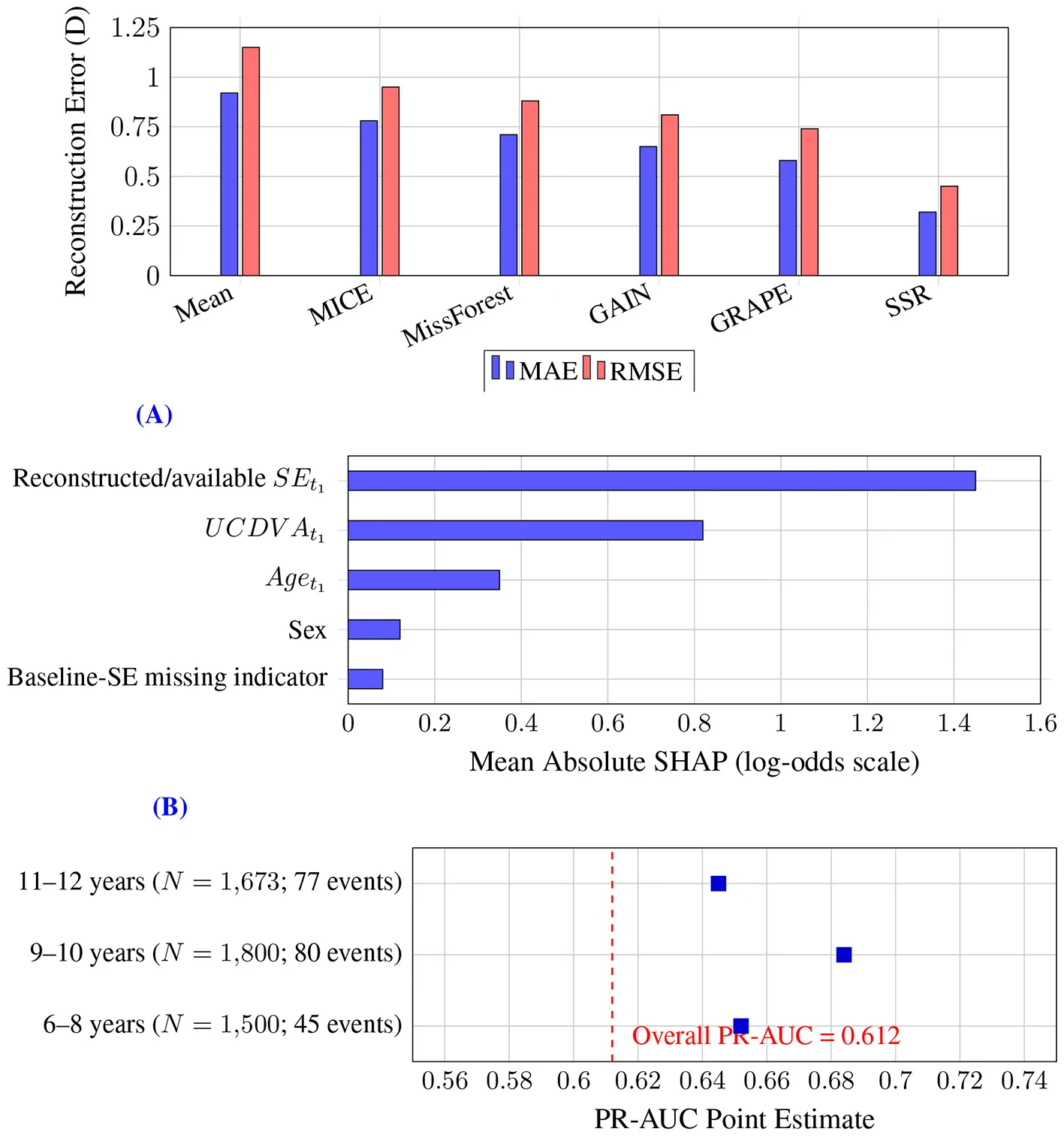
Aggregate calibration and school-level internal–external validation at 50% simulated baseline-SE missingness. **(A)** compares Brier-score summaries and does not represent a reconstructed reliability curve. **(B)** displays ROC-AUC and PR-AUC point estimates from the four leave-one-school-out iterations. School A–D are anonymized identifiers for the participating schools.

We summarized TreeSHAP attribution magnitudes for the held-out XGBoost branch (22, 23). This component-level analysis is not a global explanation of SSR-Stacking. Reconstructed or available baseline SE had the largest reported mean absolute SHAP value (1.45), followed by UCDVA (0.82), age (0.35), sex (0.12), and the baseline-SE missing indicator (0.08) (Table 6, Panel B; Figure 5B). These log-odds attribution magnitudes do not indicate direction, distribution, or causality. A beeswarm plot is not shown because the individual held-out SHAP values and corresponding feature values were not retained in the aggregate reporting export.

### 3.4 Reliability, efficiency, and summary

The revised age-stratified analysis divided the 4,973 students into 6–8, 9–10, and 11–12 year brackets to retain sufficient positive-event counts. ROC-AUC ranged from 0.876 to 0.892, PR-AUC from 0.645 to 0.684, Recall from 0.8052 to 0.8222, and Specificity from 0.9849 to 0.9855 (Table 7A, Figure 4C). Point estimates were broadly similar across age groups, and the subgroup confusion-matrix totals reproduced the overall Recall of 0.8119 and Specificity of 0.9851. Confidence intervals are not shown because subgroup bootstrap limits were unavailable.

**Table 7:** Age-stratified performance, school-level internal–external validation, and outer-fold composition.

Panel A. Age subgroup analysis							
Age bracket	*N*	Positives	Prevalence	ROC-AUC	PR-AUC	Recall	Specificity
6–8 years	1,500	45	3.00%	0.885	0.652	0.8222	0.9849
9–10 years	1,800	80	4.44%	0.892	0.684	0.8125	0.9855
11–12 years	1,673	77	4.60%	0.876	0.645	0.8052	0.9850
**Overall cohort**	**4,973**	**202**	**4.06%**	–	–	**0.8119**	**0.9851**
**Panel B. Leave-one-school-out internal–external validation**							
**Test school**	*N*	**Positives**	**Prevalence**	**ROC-AUC**	**PR-AUC**	**Recall**	**Specificity**
School A	1,200	48	4.00%	0.895	0.672	0.8125	0.9852
School B	1,500	62	4.13%	0.882	0.695	0.8065	0.9847
School C	1,300	55	4.23%	0.875	0.655	0.8182	0.9855
School D	973	37	3.80%	0.902	0.688	0.8108	0.9850
**Overall cohort**	**4,973**	**202**	**4.06%**	–	–	**0.8119**	**0.9851**
**Panel C. Stratified classroom-grouped outer-fold composition**							
**Fold**		**Training *N***	**Training positives**	**Test classrooms**	**Test** *N*	**Test positives**	**Test prevalence**
Fold 1		3,978	162	25	995	40	4.02%
Fold 2		3,978	162	25	995	40	4.02%
Fold 3		3,979	161	25	994	41	4.12%
Fold 4		3,979	161	25	994	41	4.12%
Fold 5		3,978	162	24	995	40	4.02%
**Pooled held-out folds**		–	–	**124**	**4,973**	**202**	**4.06%**

School A–D are anonymized identifiers. Each school row represents an independently held-out test institution, with all preprocessing and model fitting restricted to the remaining development schools. Panel C was generated with StratifiedGroupKFold(n_splits=5, shuffle=True, random_state=42) using classroom identifiers. Training sets overlap across rotated folds and are therefore not pooled; each student and classroom occurs exactly once in the pooled held-out rows. Bold values identify the overall-cohort summaries in Panels A and B and the pooled held-out summary in Panel C.

#### 3.4.1 School-level internal–external validation

Within-region generalizability was assessed by holding out one school at a time while fitting standardization, graph construction, reconstruction, hyperparameter optimization, stacking, and threshold selection exclusively on the remaining schools. Performance varied across held-out schools, with ROC-AUC values of 0.875–0.902 and PR-AUC values of 0.655–0.695, while Recall remained between 0.8065 and 0.8182 and Specificity between 0.9847 and 0.9855 (Table 7B, Figure 5B). School A–D are anonymized identifiers for the four participating schools.

#### 3.4.2 Outer-fold composition

The five classroom-grouped held-out folds contained 994–995 students, 24–25 classrooms, and 40–41 positive outcomes each. Their positive prevalence ranged from 4.02% to 4.12%, and concatenating the five non-overlapping held-out folds reproduced the full cohort of 4,973 students and 202 positive outcomes (Table 7C).

#### 3.4.3 Threshold selection

**Figure 6:**
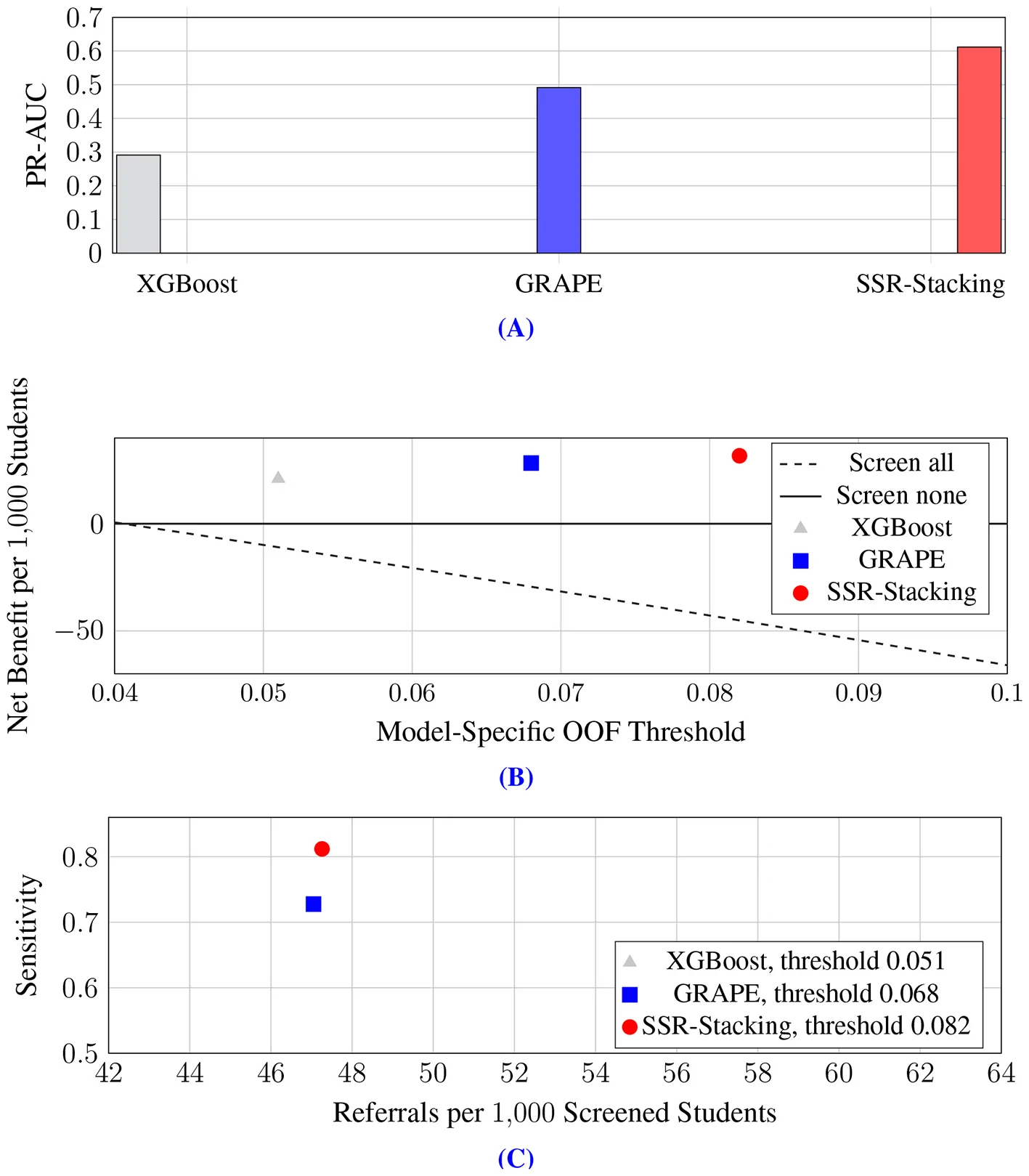
Aggregate discrimination and operating-point utility summaries. **(A)** compares reported PR-AUC estimates and is not an empirical precision–recall curve. **(B)** reports exact net benefit at each model's selected OOF threshold, calculated from the reported confusion matrix; isolated model points do not constitute full decision-curve analysis. **(C)** shows the exact referral burden and sensitivity at the same selected thresholds, with no interpolation between operating points.

The reported SSR-Stacking threshold of 0.082 is the median of fold-specific thresholds selected from development-fold OOF predictions and applied without modification to the corresponding held-out folds. At the reported operating thresholds, net benefit per 1,000 students was 20.98 for XGBoost, 28.28 for GRAPE, and 31.70 for SSR-Stacking. The corresponding referral burdens were 59.12, 47.05, and 47.26 per 1,000 students, respectively (Figures 6B, C). These are isolated operating-point summaries rather than complete decision or referral-burden curves.

The reported aggregate calibration indices for SSR-Stacking were a Brier score of 0.0268, calibration intercept of 0.0215, calibration slope of 0.9812, and observed-to-expected ratio of 0.990 (Table 8, Figure 5A). An empirical reliability curve is not reconstructed because bin-level predicted and observed probabilities were unavailable in the aggregate reporting export.

**Table 8:** Threshold and calibration summary under 50% baseline-SE missingness.

Model	Threshold	TP	FP	TN	FN	Brier score	Cal. intercept	Cal. slope	O/E
XGBoost	0.051	114	180	4,591	88	0.0385	0.1120	0.8120	–
GRAPE	0.068	147	87	4,684	55	0.0312	0.0540	0.9120	–
**SSR-stacking**	**0.082**	**164**	**71**	**4,700**	**38**	**0.0268**	**0.0215**	**0.9812**	**0.990**

Thresholds were selected from training-fold OOF predictions and applied to held-out folds. The displayed SSR-Stacking threshold is the median across outer folds. Calibration intercept and slope have ideal values of 0 and 1, respectively. O/E denotes the observed-to-expected event ratio. Bold values indicate the best performance among evaluated models/proposed framework.

#### 3.4.4 Aggregate validation graphics

To avoid inferring student-level distributions from summary statistics, the additional validation figures display only quantities that correspond directly to the reported tables or can be calculated exactly from the reported thresholds and confusion matrices. Bland–Altman scatter, SHAP beeswarm, empirical precision–recall curves, and full decision curves require paired prediction-level values and are not reconstructed from target summary metrics.

Computational efficiency. To describe computational requirements in the reported hardware environment, we measured the training and inference latency of the proposed framework (Table 9).

**Table 9:** Computational efficiency metrics (*N* = 4, 973).

Phase	Total time	Per-sample latency	Memory usage
Graph construction	14.52 s	2.92 ms	124 MB
SSR-inference	8.12 s	1.63 ms	256 MB
Total prediction	32.40 s	6.51 ms	412 MB

Despite the increased complexity of the GNN-based architecture, the measured inference latency was under 7ms per student in the reported hardware environment, supporting further assessment of computational feasibility; this benchmark does not establish clinical deployment readiness.

Summary of experimental findings. In summary, the revised experiments provide an internal classroom-grouped assessment of SSR-Stacking across a baseline-SE masking gradient. At the primary 50% masking condition, the framework achieved a ROC-AUC of 0.9184 (95% CI: 0.9062–0.9306), PR-AUC of 0.6120, Recall of 0.8119, Specificity of 0.9851, and F1-score of 0.7506 at a natural outcome prevalence of 4.06%. SSR reconstruction yielded an MAE of 0.32 D and RMSE of 0.45 D. The XGBoost component-level SHAP summary ranked reconstructed or available baseline SE first. Aggregate calibration, age-stratified, and leave-one-school-out point estimates did not reveal a clear inconsistency with the primary internal results, but these analyses do not replace natural-missingness or independent external validation.

## 4 Discussion

### 4.1 Summary of key findings

This study developed and internally evaluated **SSR-Stacking**, a graph-augmented ensemble framework designed to classify final-follow-up high-myopia status under simulated baseline-SE missingness. At 50% simulated baseline-SE missingness, the revised classroom-grouped evaluation yielded a ROC-AUC of 0.9184 (95% CI: 0.9062–0.9306), PR-AUC of 0.6120, Recall of 0.8119, Specificity of 0.9851, balanced accuracy of 0.8985, and F1-score of 0.7506. The internal results suggest that classroom-level relational embeddings may complement individual measurements when baseline SE is masked; this association does not establish a causal influence of classmates or classroom exposure.

### 4.2 Relational compensation: comparison with standalone ML

Traditional models such as XGBoost and Random Forest treat students as independent observations and do not use classroom adjacency. In the present benchmark (Table 3), these models showed a larger reduction in sensitivity when baseline SE was masked.

In contrast, **SSR-stacking** operates on a **Social-Environment Graph**. Our analysis revealed an ICC of 0.24 and a Global Moran's I of 0.21 for SE progression within classroom clusters, indicating classroom-level statistical clustering. Classroom membership may reflect a mixture of shared exposures, demographic composition, selection, and unmeasured contextual factors; the present observational analysis cannot separate these explanations. At 50% masking, SSR-Stacking increased Recall from 0.7277 for GRAPE to 0.8119 and PR-AUC from 0.4915 to 0.6120. At 80% masking, its Recall remained 0.7327 and PR-AUC 0.5322, compared with 0.6337 and 0.3810 for GRAPE. The held-out reconstruction analysis further showed lower error for SSR than for the evaluated imputation baselines (MAE 0.32 D versus 0.58 D for GRAPE), supporting reconstruction fidelity as one plausible contributor to the classification results.

### 4.3 Complementary Contributions of SSR and Stacking

The revised ablation showed that adding SSR to GATv2 increased PR-AUC by 0.1085, whereas adding stacking increased PR-AUC by 0.0610. The full framework improved PR-AUC by 0.1835 and Recall by 12.87 percentage points relative to GATv2 only. The reported Brier score of 0.0268, calibration intercept of 0.0215, calibration slope of 0.9812, and observed-to-expected ratio of 0.990 did not indicate severe systematic probability miscalibration; a reliability curve is not inferred from these aggregate values.

### 4.4 Comparison with existing literature

Many myopia prediction studies have emphasized discrimination or accuracy in datasets with relatively complete clinical measurements (24–29). Routine school screenings may instead contain absence- and measurement-related gaps. Reviews of AI for high myopia similarly identify missing-data robustness, interpretability, and transportability as barriers to clinical translation (30, 31).

Our work evaluates relational modeling as an extension to conventional imputation-based analysis. Unlike ensemble pipelines based only on non-relational imputation (5, 6), **SSR-Stacking** uses classroom adjacency during self-supervised feature reconstruction. This design is consistent with broader developments in relational learning, but its value in public-health surveillance requires external evaluation (32–34).

### 4.5 Scope of the missingness evaluation

Current evaluations of **SSR-Stacking** are limited to randomly simulated masking of baseline SE. From a clinical perspective, data missingness in school screenings may be Missing Completely at Random (MCAR), Missing at Random (MAR), or Missing Not at Random (MNAR). The present random-masking experiment most closely approximates an MCAR stress test and does not establish performance under natural MAR or MNAR mechanisms.

In more challenging MNAR scenarios, such as students with severe myopia being more likely to seek private care and therefore missing school-based screenings, imputation models may introduce systematic bias. Whether classroom-level relational information reduces or amplifies such bias remains unknown. Validation using the natural missingness pattern, multivariable missingness, and visit-level dropout is therefore required before claims regarding MAR, MNAR, or real-world robustness can be made. A planned secondary analysis will return to the registered screening cohort, retain naturally incomplete *t*_1_ predictors among students with an observed *t*_2_ outcome and usable classroom information, and compare SSR-Stacking with native-missingness XGBoost, MICE, MissForest, GAIN, and GRAPE under common classroom-grouped partitions. Missingness indicators will be modeled from observed pre-index demographic, ocular, attendance, school, and classroom variables to describe associations compatible with MAR. Because MNAR cannot be established from observed records alone, robustness will additionally be examined through pattern-mixture or delta-adjustment sensitivity analyses that shift imputed SE values across clinically justified ranges and identify assumptions under which the principal conclusions change. These analyses are stated as future work and are not evidence provided by the present study.

### 4.6 Clinical implications and limitations

The revised age-stratified estimates were broadly similar across the 6–8, 9–10, and 11–12 year groups, although the limited positive-event counts within each bracket warrant cautious interpretation and do not establish age-specific clinical utility.

However, several limitations remain. First, the outcome is final-follow-up high-myopia status, and students with high myopia at the model-index visit were retained; the analysis therefore does not estimate incident conversion among baseline non-high-myopic students. Second, graph edges were defined solely by classroom affiliation. More granular contextual measurements may improve interpretation, although friendship or behavioral networks would introduce additional privacy and confounding concerns. Third, random masking of one baseline variable is a controlled stress test and does not reproduce natural multivariable missingness, visit-level dropout, covariate-dependent MAR, or outcome-related MNAR. Although variable-specific natural missingness is reported by screening wave, the final cohort was selected as a complete panel and the models were not evaluated directly on naturally incomplete records. This complete-case design may introduce selection bias and does not identify the underlying missingness mechanism. Fourth, the outcome prevalence was 4.06% (202 events), limiting precision in age and school subgroups. Fifth, repeated-seed uncertainty and paired classroom-cluster intervals were unavailable for several ablation and between-model comparisons. Sixth, the SHAP summary explains only the XGBoost branch and is neither causal nor a complete explanation of the hybrid framework. Seventh, leave-one-school-out validation involved four schools from the same regional cohort and does not replace independent geographic or temporal external validation. A future external evaluation will freeze the preprocessing parameters, trained model weights, graph-construction rules, and operating threshold before applying the framework to a non-overlapping later screening cohort and, where available, a geographically independent school-screening registry. Graphs will be constructed exclusively from classrooms within the external cohort, with no development-to-validation edges or outcome-informed retuning. External performance will be reported using prevalence, natural missingness, ROC-AUC, PR-AUC, Brier score, calibration intercept and slope, and the confusion matrix at the frozen threshold, with uncertainty calculated at the classroom or school level. Eighth, the analysis code is available only upon request rather than through a public version-controlled repository, which limits independent computational reproduction. Ninth, distribution-level analyses such as Bland–Altman scatter, SHAP beeswarm, empirical precision–recall curves, reliability curves, complete decision curves, and subgroup confidence intervals were not reconstructed from aggregate point estimates. Finally, although inference was under 7 ms per student in the reported hardware environment, this computational benchmark does not establish clinical deployment readiness.

### 4.7 Future validation priorities

The immediate methodological priority is to evaluate the frozen framework under the natural predictor-missingness patterns observed in the registered cohort and to perform explicit MNAR sensitivity analyses rather than assigning a missingness mechanism from observed data alone. The subsequent validation priority is application to temporally non-overlapping and geographically independent cohorts without model redevelopment. Together, these studies will determine whether the present internal findings persist under natural missingness, calendar-time change, institutional variation, and differences in outcome prevalence.

### 4.8 Conclusion

**SSR-Stacking** combines classroom-graph reconstruction with heterogeneous ensemble learning for final-follow-up high-myopia classification under simulated baseline-SE missingness. The classroom-grouped internal evaluation showed improved point estimates relative to the reported comparators, but the study does not establish incident-risk prediction, performance under natural MAR or MNAR mechanisms, or deployment readiness.Natural-missingness evaluation, MNAR sensitivity analysis, and validation in frozen temporally and geographically independent cohorts are required before clinical or public-health use.

## Data Availability

The raw data supporting the conclusions of this article will be made available by the authors, without undue reservation.
